# First M. E. Church, South, W. D. Bradfield, Pastor

**Published:** 1912-09

**Authors:** W. D. Bradfield

**Affiliations:** Austin, Texas


FIRST M. E. CHURCH, SOUTH,
W. D. BRADFIELD, PASTOR.

AUSTIN, TEXAS, August 20, 1912.

*Mrs. F. E. Daniel, Austin, Texas.*

DEAR MRS. DANIEL: I note with much pleasure your plan to establish a Woman's Department in the "Red Back." This noble JOURNAL for many years has filled a needed place in the medical profession, and the appearance of your department will greatly extend its usefulness.

I hail with especial pleasure this new evidence that women are to have an increasingly large place in solving the problems which so vitally affect the womanhood of our times. There are many things waiting to be said to the women of our day which only women can best say.

Wishing you great success in your worthy enterprise, I remain,

Very truly yours,

W. D. BRADFIELD.

